# Zika virus infection and implications for kidney disease

**DOI:** 10.1007/s00109-018-1692-z

**Published:** 2018-08-31

**Authors:** Donald J. Alcendor

**Affiliations:** Center for AIDS Health Disparities Research, Department of Microbiology, Immunology, and Physiology, School of Medicine, Meharry Medical College, 1005 Dr. D.B. Todd Jr. Blvd., Hubbard Hospital, 5th Floor, Rm. 5025, Nashville, TN 37208 USA

**Keywords:** Zika virus, Kidney, Glomerulus, Viruria

## Abstract

High-level and persistent viruria observed in patients infected by Zika virus (ZIKV) has been well documented. However, renal pathology in acutely infected, immunocompetent patients remains subclinical. Moreover, the long-term impact of ZIKV infection, replication, and persistence in the renal compartment of adults and infants as well as immunosuppressed patients and solid organ transplant (SOT) recipients is unknown. Mechanisms involving host and viral factors that limit or control ZIKV pathogenesis in the renal compartment are important yet unexplored. The observation that long-term viral shedding occurs in the renal compartment in the absence of clinical disease requires further investigation. In this review, I explore Zika virus-induced renal pathology in animal models, the dynamics of virus shedding in urine, virus replication in glomerular cells, ZIKV infection in human renal transplantation, and the potential impact of long-term persistent ZIKV infection in the renal compartment.

## Introduction

Zika virus (ZIKV) is a positive sense single-stranded RNA virus of the *Flaviviridae* family, which belongs to the genus *Flavivirus*. The genus *Flavivirus* also includes dengue, West Nile, Japanese encephalitis, and yellow fever viruses [1, 2]. ZIKV is transmitted to humans primarily through bites by infected *Aedes* mosquitoes [3, 4]. ZIKV was first isolated in 1947 from sentinel monkeys in the Zika Forest of Uganda during surveys conducted for yellow fever [5]. The earliest cases of ZIKV infection were identified in three patients during a jaundice outbreak in Eastern Nigeria in 1954 [6]. ZIKV infections remained low and sporadic until the outbreak in Yap Islands of Micronesia in 2007 then spreading to the Pacific Islands in 2013–2014 [7–9]. These outbreaks were found to be mild and self-limiting without adverse clinical outcomes. In 2015, ZIKV spread to Brazil, causing severe congenital disease in infants born to ZIKV-infected mothers. These impairments included microcephaly, intrauterine growth restriction, congenital contractures, fetal demise, ocular abnormalities, neurodevelopmental disorders, and Guillain-Barre syndrome [10–15]. Currently, there is no FDA-approved vaccine for preventing ZIKV infection.

Several clinical studies have demonstrated high levels of excreted infectious ZIKV in the urine of acutely infected patients [16–18]. ZIKV can be detected in the urine of both adult and infant patients and can be cultured directly from the urine samples [19, 20]. Viremia as demonstrated by quantitative reverse transcription-polymerase chain reaction (qRT-PCR) was observed 2 days after disease onset; however, there was persistent shedding of high levels of ZIKV RNA into the urine for up to 15 days after the onset of symptoms [18, 21]. Chan et al., using qRT-PCR assays targeting the ZIKV-5′-UTR, observed a relatively low viral load of 2.35 × 10^2^ in urine samples from human patients, with a range of 1.25 × 10^2^–5.56 × 10^2^ [22]. However, Gourinat et al. examined urine samples from six ZIKV-infected patients from New Caledonia and found high viral loads with a range of 0.7–220 × 10^6^ copies/ml. They also reported the use of urine samples for diagnostics because viral titers were found to be higher and present for a longer duration in the urine than in the serum [19]. In a study by Terzian et al., they revealed long-term intermittent viruria in ZIKV-infected pregnant women for up to 7 months after the onset of symptoms [23]. Interestingly, viruria was no longer detected post-partum, suggesting that a reservoir for ZIKV was maintained in the fetal tissue rather than in the infected mother [23]. The pathological effects and long-term consequences of ZIKV shedding in the urinary tract are unknown. The subclinical nature of viral shedding in the renal compartment is also largely unknown. The effects of long-term viruria in pregnancy and fetal outcomes have not been reported. To the best of my knowledge, no obvious renal disease or abnormalities have been observed to date in ZIKV-infected patients, and no comprehensive studies have been performed on renal biopsies from patients infected with ZIKV. Therefore, a comprehensive examination of cellular targets for ZIKV replication in the renal compartment is required.

In 2017, I published a study reporting that the high viral burden in the renal compartment correlated directly with ZIKV infection of glomerular parenchymal cells in vitro that includes podocytes, glomerular endothelial cells, and mesangial cells [24]. These cells are highly permissive to ZIKV and likely serve as ZIKV amplification reservoirs, resulting in long-term persistent viral shedding in the urine [24]. A report by Chen et al. showed that ZIKV infection of renal proximal tubular epithelial cells (hRPTEpiCs) resulted in prolonged persistent infection and ZIKV cytopathology in immunodeficient mice in vivo as well as in immortalized (hRPTEpiCs) [25].

In this review, I examine the renal pathology found in ZIKV-infected murine and non-human primate models, in vitro ZIKV infection of the glomerular cells, the potential long-term effects of ZIKV infection of the kidney, and the potential impact of ZIKV infection on renal transplantation. My goal is to provide a literature review of published findings on ZIKV pathogenesis in the kidney and highlight the urgent need for future studies in this area.

## ZIKV infection and renal pathology in animal models

### Mouse models

Mouse models for ZIKV infection have been used extensively because they are relatively inexpensive, have short gestation times, produce large litters, and are suitable for vaccine studies and therapy evaluations. However, most susceptible mouse strains are deficient in interferon (IFN) responses and may display pathologies that are not normally observed in immunocompetent humans [26]. There are a limited number of studies that used murine models to examine the renal pathology associated ZIKV infection. A recent report by Chen et al. showed that ZIKV infection of renal proximal tubular epithelial cells resulted in prolonged persistence and ZIKV cytopathology in immunodeficient mice in vivo [25]. In this study, the authors used AG6 mice, which are C57BL/6 mice deficient in type I and type II IFN receptors, as a model for in vivo ZIKV renal infection. The authors detected ZIKV infection in both the glomeruli and tubules of the AG6 mice on day 7 post-infection with the SZ01 Asian strain of ZIKV using in situ hybridization for ZIKV RNA [25]. The degree of infection was higher in the glomeruli than in the tubules. This observation suggests that the initial ZIKV penetrance occurred in the glomeruli, followed by dissemination to the tubules during renal filtration [25]. The authors also found ZIKV-induced swelling of the mouse kidneys and apoptosis of renal cells as indicated by caspase-3 induction, as well as the dysregulation of IFN signaling and antiviral response genes [25]. Ferreira et al. observed ZIKV infection in the blood plasma, spleen, kidney, and brain of Swiss albino mice infected with the African (MR766) and Brazilian strains of ZIKV [26]. ZIKV RNA reached its peak titer of 7 × 10^4^ copies/10^5^ cells in the kidney 3 days after infection, as determined by qRT-PCR performed on RNA extracted from kidney homogenates [26]. Zmurhko et al. detected high levels of ZIKV RNA in the spleen, liver, and kidneys of AG129 mice, which are 129/Sv mice deficient in IFN-α/β and IFN-γ receptors [27]. Aliota et al. reported that infection of AG129 mice with the French Polynesian strain of ZIKV resulted in high viral loads in multiple organs including the kidneys, liver, and spleen 7 days after infection [28]. ZIKV titers observed in the kidney as determined by qRT-PCR ranged from 10^6^ to 10^9^ copies/g of tissue. However, no significant histopathology or obvious tissue damage associated with ZIKV infection was observed [28]. A study by Chan et al., employing a mouse model for ZIKV infection of dexamethasone-immunosuppressed animals, demonstrated that mice whose kidneys were infected by the Puerto Rico ZIKV strain PRVABC59 suffered from acute tubulitis and inflammatory exudation in tubular lumens with interstitial inflammation [29]. Studies using immunosuppressed animals by Lazear et al., and Rossi et al., also showed high ZIKV viral loads in the spleen, liver, kidneys, serum, testes, brain, and spinal cord of infected animals [30, 31].

### Non-human primate models

Non-human primates are natural hosts for ZIKV and have been used to mimic ZIKV infection in humans. Non-human primates (rhesus, cynomolgus, and pig-tailed macaques) are highly permissive for multiple strains of ZIKV, can be infected by multiple routes including vector-borne transmission, and are virologically and clinically similar to humans. Dudley et al. showed that rhesus macaques infected with ZIKV via mosquito bites exhibited altered tissue tropism and replication kinetics [32]. In macaques infected via mosquito bites, the authors observed a delay in the appearance of peak viral loads, and viral dissemination was limited to hemolymphatic tissues, female reproductive tract tissues, kidney, and liver [32]. The dissemination pattern was similar to that observed in asymptomatic humans infected via mosquito bites [32]. A study by Hirsch et al. uncovered viruria and viral RNA in the kidney and bladder of ZIKV-infected rhesus macaques 7 days after infection, although the virus was not detected in the testes or prostate by this time [33].

Additionally, Hirsch et al., Newman et al., Kublin et al., and Osuna et al. have published comprehensive reviews on the use of non-human primates to model ZIKV infection [33–36]. However, to the best of my knowledge, the non-human primate studies described in these reviews did not extensively examine the glomerular tissue during acute and convalescent phases of the disease after ZIKV infection.

## ZIKV infection of human glomerular parenchymal cells

**Fig. 1:**
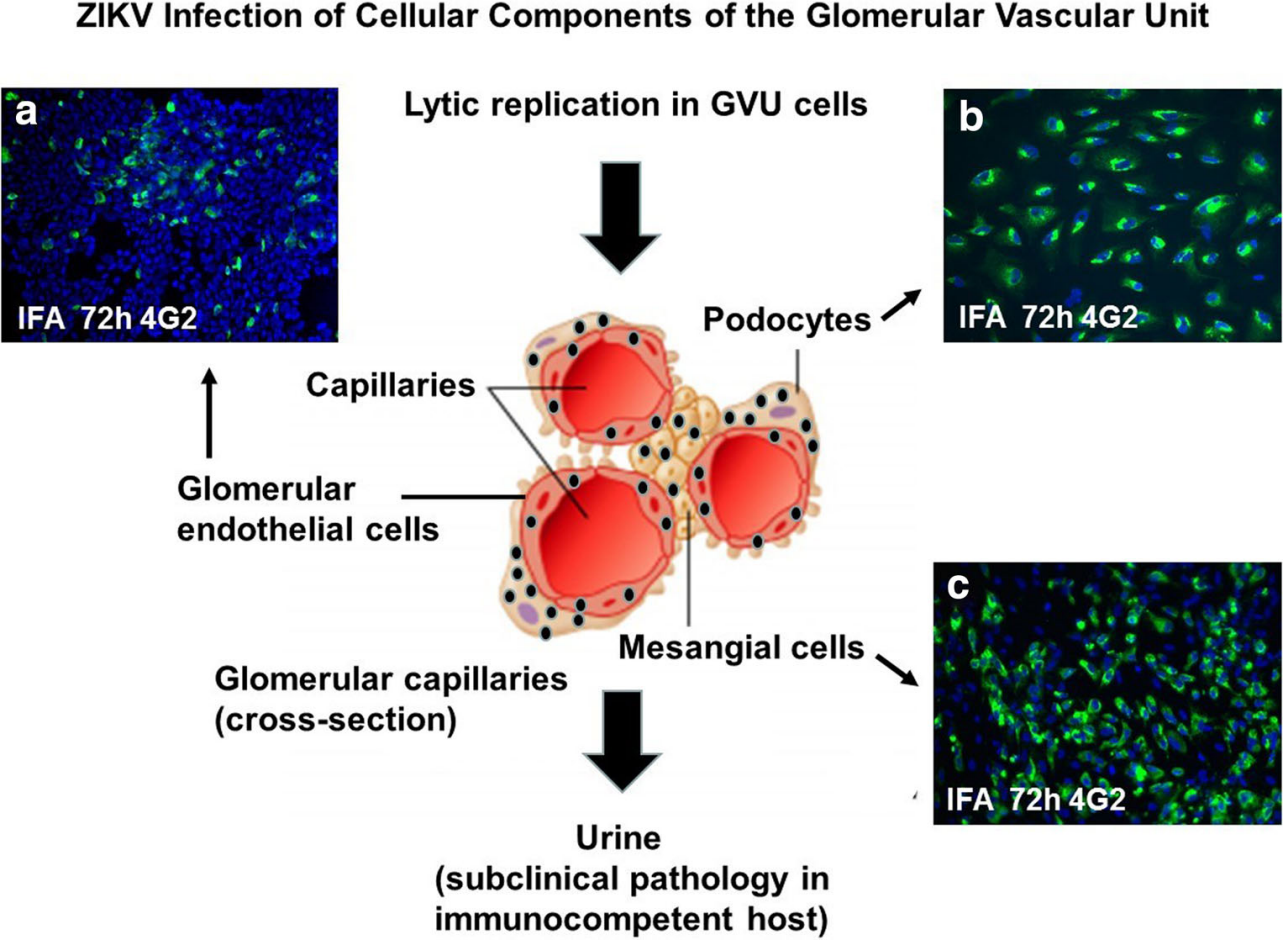
ZIKV lytic replication in the cellular components of the glomerular vascular unit (GVU). **a** Immunofluorescence antibody assay (IFA) using the flavivirus 4G-2 antibody. ZIKV lytic replication was observed in primary human glomerular endothelial cells 72 h post-infection. **b** IFA using the 4G-2 antibody showing ZIKV lytic replication in human glomerular podocytes. **c** IFA using the 4G-2 antibody showing ZIKV lytic replication in primary human mesangial cells. Cells positive for ZIKV stained green (FITC). ZIKV is depicted by black dots. All images were taken on a Nikon TE2000S microscope mounted with a charge-coupled device (CCD) camera at × 200 magnification. For the fluorescent images, 4′,6-diamidino-2-phenylindole (DAPI) was used to stain the nuclei blue

ZIKV has been shown to replicate in glomerular parenchymal cells of the adult human kidney. Chan et al. showed that human embryonic kidney (HEK) cells are permissive for ZIKV, which may explain the high viral loads in urine of ZIKV-infected patients [37]. However, the high viral loads and expression level of ZIKV-NS1 protein in the absence of ZIKV cytopathology suggest that the kidney could be a preferred site of persistent ZIKV replication [37]. I have determined that human glomerular podocytes, renal glomerular endothelial cells, and mesangial cells, collectively referred to as the glomerular vascular unit (GVU), are permissive for ZIKV infection and lytic replication in vitro (Fig. 1) [24]. In this study, I concluded that ZIKV infection of podocytes, glomerular endothelial cells, and mesangial cells likely contributes to the high-level persistent viruria observed in ZIKV-infected patients [24]. If the same high degree of lytic infection I observed in vitro after ZIKV infection also occurred in ZIKV-infected patients, it would cause podocyte destruction, severe proteinuria, and ultimately end-stage renal disease [38–40] (Fig. 1). I also observed the highest infection burden in podocytes and that the induction of the pro-inflammatory cytokine RANTES correlated with virus replication in the glomerular cells [24].

**Fig. 2:**
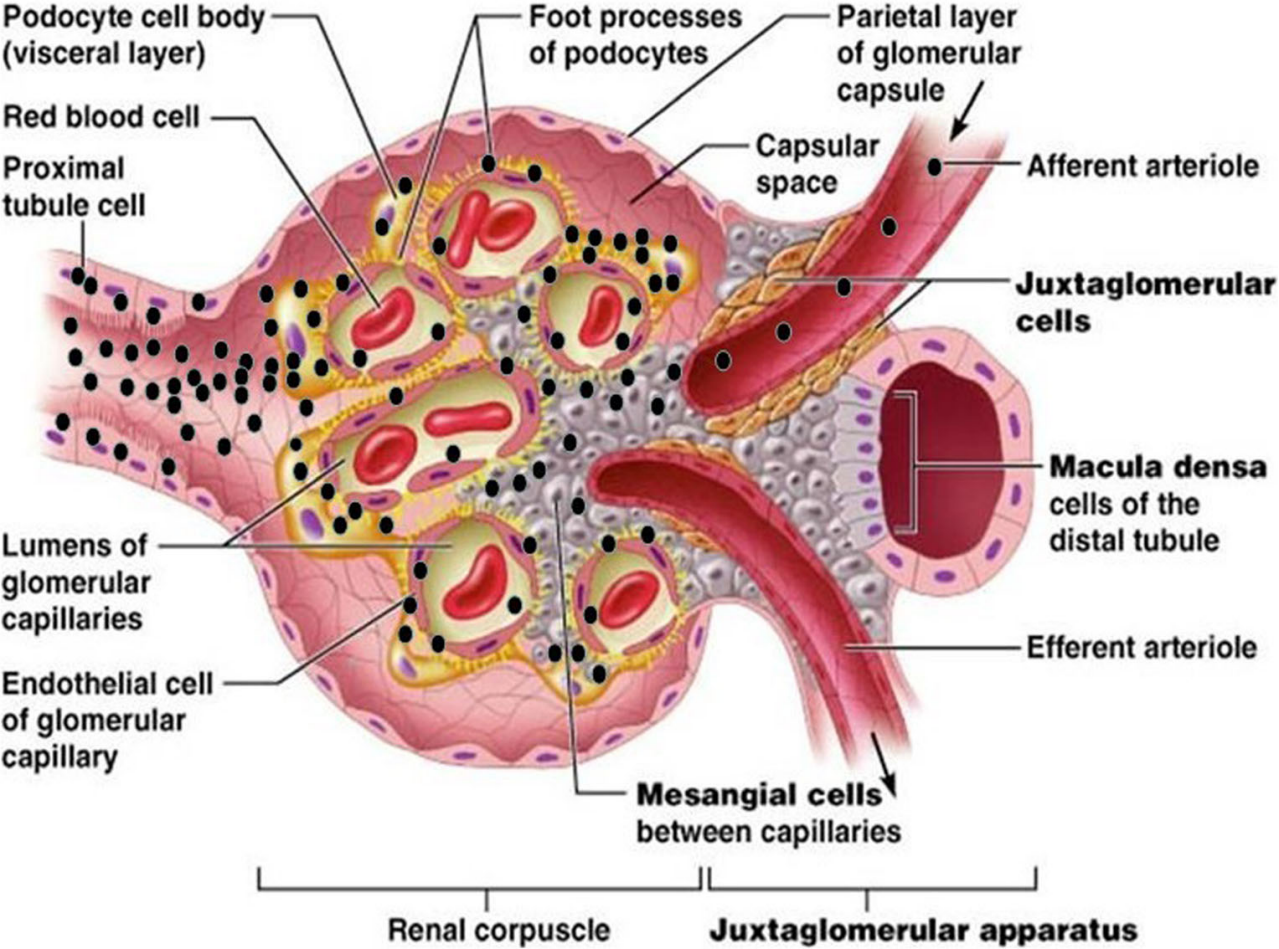
Hypothetical model for ZIKV dissemination in the glomerulus of the human kidney. A hypothetical model for ZIKV entry and presence in the glomerulus (modified with permission from Pearson Education Inc. 2013 [unpublished data]). ZIKV, depicted by black dots, enters the bloodstream via a bite by an infected mosquito. In the viremic phase of the infection, blood containing ZIKV enters the glomerulus via the afferent arterioles and glomerular capillaries, leading to infection of the renal corpuscle and subsequently the glomerular endothelial cells in the kidney. The virus spreads from the infected glomerular endothelial cells to the glomerular parenchyma. Mesangial cells, podocytes, and renal proximal tubular epithelial cells (hRPTEpiCs) become highly exposed to infectious ZIKV. Podocytes, mesangial cells, and proximal tubular cells are highly permissive to ZIKV and likely serve as ZIKV amplification reservoirs in the glomerulus, resulting in high-level persistent viruria

My current hypothetical model for ZIKV dissemination in the glomerulus of human kidney begins with virus access via the bloodstream after a bite by an infected mosquito (Fig. 2). ZIKV then enters the glomerulus via the afferent arterioles and glomerular capillaries, leading to infection of the renal corpuscle and subsequently the glomerular endothelial cells (Fig. 2). The virus spreads from the infected glomerular endothelial cells to the glomerular parenchyma. Mesangial cells, podocytes, and hRPTEpiCs then become highly exposed to infectious ZIKV (Fig. 2). Podocytes, mesangial cells, and hRPTEpiCs are highly permissive for ZIKV and likely serve as ZIKV amplification reservoirs in the glomerulus, resulting in high-level persistent viruria (Fig. 2), [24].

On the other hand, findings by Peralta-Aros et al. suggest that ZIKV infection of the renal compartment may induce host cell factors that support renal function [41]. In this study, the authors showed that ZIKV infection induced prolonged remissions in children with idiopathic nephrotic syndrome (INS) [41]. This phenomenon occurred in only two patients, who achieved complete remission of the disease after acute ZIKV infection. Therefore, the authors concluded that disease remission could be a random event unrelated ZIKV infection [41]. Nonetheless, the underlying mechanisms that may support a direct role for ZIKV infection in INS remissions warrant further investigation.

## Potential long-term impact of ZIKV infection in the kidney

**Fig. 3:**
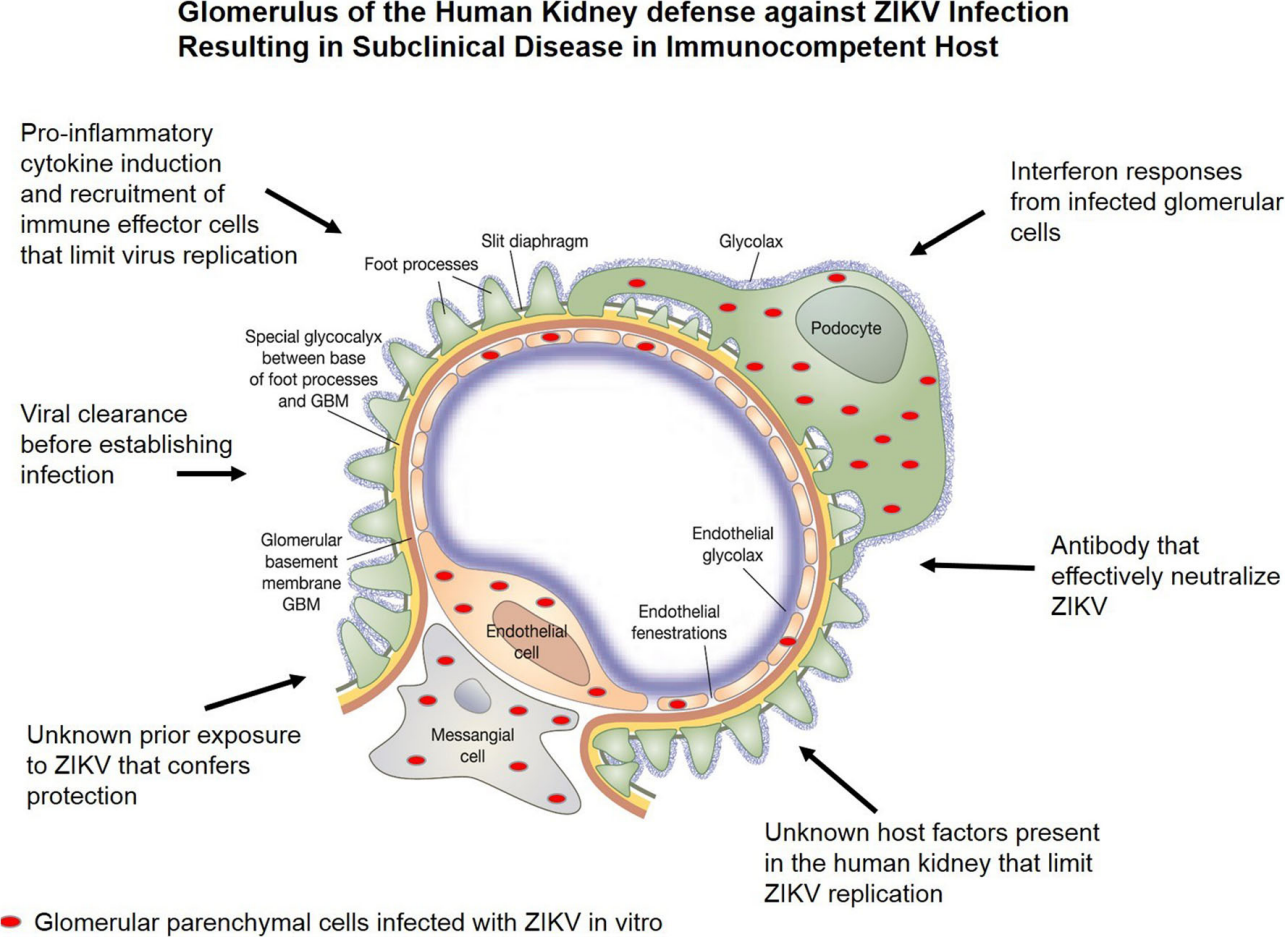
Proposed defense mechanisms in the human kidney against ZIKV infection that result in a subclinical disease in immunocompetent hosts. A model for ZIKV dissemination in the glomerular vascular unit (GVU) and proposed mechanisms that result in subclinical disease in immunocompetent individuals. ZIKV is depicted by red ovals

ZIKV cytopathology in glomerular cells is likely controlled by immune surveillance because patients recover from the infection and viruria subsides over time. There are likely host immune factors that limit viral infection and dissemination in the glomerulus, resulting in a self-limiting disease that remains subclinical throughout the disease course (Fig. 3). However, long-term effects of ZIKV replication in the glomeruli of adults and infants as well as immunocompromised patients are unknown. Obtaining fresh renal tissue from ZIKV-infected patients with acute disease has been difficult. However, using in situ hybridization (ISH) and electron microscopy, Chimelli et al. performed postmortem analysis on human fetal autopsy tissue of 10 pregnancies (stillborn or newborn babies who died in the first 37 h of life) from 10 ZIKV-infected mothers in Brazil [42]. Upon examination of renal tissue, they observed the presence of ZIKV infection mainly in the renal tubular cells in four out of eight fetal kidneys examined [42]. There is a great need for more comprehensive studies using renal biopsies or archived renal tissues from ZIKV-infected adults and infants with congenital Zika syndrome (CZS) to determine viral dissemination patterns and possible long-term persistence of viral genomes within the glomeruli. Studies using animal models to examine long-term exposure of glomerular cells to ZIKV in vivo in the presence and absence of immunosuppression are essential. Careful examination of the renal tissues from these animals to detect long-term persistence of viral genomes is also crucial. Identification of biomarkers in animals and humans infected with ZIKV is also needed. These biomarkers can be used to detect ZIKV infection and to monitor glomerular function.

I am also aware that acute renal disease has not been observed in ZIKV-infected patients. Although I have observed full lytic replication of ZIKV in primary human glomerular cells in vitro, I have not encountered reports on immunocompetent patients having renal disease that is directly associated with ZIKV infection. However, a recent study by Costa-Monteiro et al. reported on the development of neurogenic bladder in 21 pediatric patients with CZS [43]. All 21 patients clinically presented with an overactive bladder, with reduced bladder capacity and elevated detrusor pressure [43]. All patients were tested and confirmed to have neurogenic bladder. If left untreated, it could lead to recurrent urinary tract infections, urinary incontinence, and ultimately chronic and end-stage renal diseases. To the best of my knowledge, this is the first report that confirms a diagnosis of neurogenic bladder in infants with CZS [43]. The high incidence of neurogenic bladder observed in this study implies that all infants born with CZS should be screened for this condition in order to provide early treatment interventions to improve long-term clinical outcomes. In immunocompetent adults, there are likely underlying host defense mechanisms that limit ZIKV pathology in the glomerulus (Fig. 3). These currently unidentified host factors may limit ZIKV pathogenesis in the absence of immunosuppression and could be important for the development of novel antiviral strategies to prevent ZIKV transmission during pregnancy.

## ZIKV infection and implications for renal transplantation

The risk of ZIKV infection in solid organ transplant (SOT) patients has not been fully examined. Disease severity associated with ZIKV infection in the context of immunosuppression for allograft maintenance is unknown. The impact of ZIKV-associated disease on allograft function has not been reported. Nogueira et al. reported on the first series of cases of ZIKV infection in SOT patients who tested positive for ZIKV and negative for other arboviruses, including dengue virus and chikungunya virus [44]. In this study, ZIKV infection in both liver and renal transplant patients resulted in clinical complications, most notably bacterial infections [44]. While none of the ZIKV-infected patients presented with a rash, conjunctivitis, or other neurological symptoms, three of the four patients examined were anemic and all of them had thrombocytopenia [44]. These patients presented at admission with fever, myalgia, and adynamia along with signs of acute liver or renal damage [44]. Unfortunately, the sampling for this study was small and is not sufficient to illustrate the impact of ZIKV infection in SOT patients. Therefore, future studies with larger sample sizes are required. The incidence of ZIKV pathology in SOT patients will likely increase over time. Mandatory screening of allograft donors and recipients from ZIKV endemic regions may be warranted to prevent ZIKV infection during organ transplantation.

## Conclusion

The cause of subclinical pathology coupled with high-level and persistent viruria during acute ZIKV infection in immunocompetent patients is unclear [45, 46]. I propose that ZIKV infection may specifically induce proinflammatory cytokines that limit virus replication and glomerular injury (Fig. 3). The IFN responses induced by ZIKV infection in the glomerular microenvironment may be sufficient to limit or control virus dissemination (Fig. 3). I am aware that ZIKV only replicates effectively in the peripheral organs of mice when type I IFN signaling is interrupted [47, 48]. There could be mechanisms in the glomeruli in humans that circumvent this restriction. There may also be pre-existing or cross-neutralizing antibodies that effectively neutralize ZIKV in the kidney over time [49] (Fig. 3). It is also possible that some unidentified host factors in the human kidney limit ZIKV lytic replication and prevents overt clinical disease (Fig. 3). Additionally, some individuals may have experienced prior subclinical exposure to ZIKV and have developed immunity against the virus (Fig. 3). Lastly, it is possible that the glomeruli in humans have acquired some form of adaptation to facilitate viral clearance before ZIKV infection and clinical disease can be established (Fig. 3).

## References

[1] Faye O, Freire CCM, Iamarino A, Faye O, de Oliveira JVC, Diallo M, Zanotto PMA, Sall AA (2014). Molecular evolution of Zika virus during its emergence in the 20th century. PLoS Negl Trop Dis.

[2] Hayes EB (2009). Zika virus outside Africa. Emerg Infect Dis.

[3] Tetro JA (2016). Zika and microcephaly: causation, correlation, or coincidence?. Microbes Infect.

[4] Oster AM, Brooks JT, Stryker JE, Kachur RE, Mead P, Pesik NT, Petersen LR (2016). Interim guidelines for prevention of sexual transmission of Zika virus—United States. MMWR Morb Mortal Wkly Rep.

[5] Dick GW, Kitchen SF, Haddow AJ (1952). Zika virus: isolations and serological specificity. Trans R Soc Trop Med Hyg.

[6] Macnamara FN (1954). Zika virus: a report on three cases of human infection during an epidemic of jaundice in Nigeria. Trans R Soc Trop Med Hyg.

[7] Lanciotti RS, Kosoy OL, Laven JJ, Velez JO, Lambert AJ, Johnson AJ, Stanfield SM, Duffy MR (2008). Genetic and serologic properties of Zika virus associated with an epidemic, Yap State, Micronesia. Emerg Infect Dis.

[8] Cao-Lormeau VM, Roche C, Teissier A (2013). Zika virus, French polynesia, South Pacific. Emerg Infect Dis.

[9] Slavov SN, Otaguiri KK, Kashima S (2016). Overview of Zika virus (ZIKV) infection in regards to the Brazilian epidemic. Braz J Med Biol Res.

[10] Zanluca C, Melo VC, Mosimann AL (2015). First report of autochthonous transmission of Zika virus in Brazil. Mem Inst Oswaldo Cruz.

[11] Musso D (2015). Zika virus transmission from French Polynesia to Brazil. Emerg Infect Dis.

[12] Schuler-Faccini L, Ribeiro EM, Feitosa IM (2016). Possible association between Zika virus infection and microcephaly—Brazil. MMWR Morb Mortal Wkly Rep.

[13] Marrs C, Olson G, Saade G (2016). Zika virus and pregnancy: a review of the literature and clinical considerations. Am J Perinatol.

[14] Garcia E, Yactayo S, Nishino K (2016). Zika virus infection: global update on epidemiology and potentially associated clinical manifestations. Wkly Epidemiol Rec/Heal Sect Secr Leag Nations.

[15] McGrogan A, Madle GC, Seaman HE, de Vries CS (2009). The epidemiology of Guillain-Barre syndrome worldwide. A systematic literature review. Neuroepidemiology.

[16] Zhang FC, Li XF, Deng YQ, Tong YG, Qin CF (2016). Excretion of infectious Zika virus in urine. Lancet Infect Dis.

[17] Rozé B, Najioullah F, Fergé JL, GBS Zika Working Group (2016). Zika virus detection in urine from patients with Guillain-Barré syndrome on Martinique, January 2016. Euro Surveill.

[18] Campos Rde M, Cirne-Santos C, Meira GL (2016). Prolonged detection of Zika virus RNA in urine samples during the ongoing Zika virus epidemic in Brazil. J Clin Virol.

[19] Gourinat AC, O'Connor O, Calvez E (2015). Detection of Zika virus in urine. Emerg Infect Dis.

[20] Fonseca K, Meatherall B, Zarra D (2014). First case of Zika virus infection in a returning Canadian traveler. Am J Trop Med Hyg.

[21] Bingham AM, Cone M, Mock V, Heberlein-Larson L, Stanek D, Blackmore C, Likos A (2016). Comparison of test results for Zika virus RNA in urine, serum, and saliva specimens from persons with travel-associated Zika virus disease—Florida, 2016. MMWR Morb Mortal Wkly Rep.

[22] Chan JF, Yip CC, Tee KM (2017). Improved detection of Zika virus RNA in human and animal specimens by a novel, highly sensitive and specific real-time RT-PCR assay targeting the 5′-untranslated region of Zika virus. Tropical Med Int Health.

[23] Terzian ACB, Estofolete CF, Alves da Silva R, Vaz-Oliani DCM, Oliani AH, Brandão de Mattos CC, Carlos de Mattos L, Rahal P, Nogueira ML (2017). Long-term viruria in Zika virus-infected pregnant women, Brazil, 2016. Emerg Infect Dis.

[24] Alcendor DJ (2017). Zika virus infection of the human glomerular cells: implications for viral reservoirs and renal pathogenesis. J Infect Dis.

[25] Chen J, Yang YF, Chen J (2017). Zika virus infects renal proximal tubular epithelial cells with prolonged persistency and cytopathic effects. Emerg Microbes Infect.

[26] Ferreira AC, Zaverucha-do-Valle C, Reis PA (2017). Sofosbuvir protects Zika virus-infected mice from mortality, preventing short- and long-term sequelae. Sci Rep.

[27] Zmurko J, Marques RE, Schols D, Verbeken E, Kaptein SJF, Neyts J (2016). The viral polymerase inhibitor 7-deaza-2'-C-methyladenosine is a potent inhibitor of in vitro Zika virus replication and delays disease progression in a robust mouse infection model. PLoS Negl Trop Dis.

[28] Aliota MT, Caine EA, Walker EC (2016). Characterization of lethal Zika virus infection in AG129 mice. PLoS Negl Trop Dis.

[29] Chan JF, Zhang AJ, Chan CC (2016). Zika virus infection in dexamethasone-immunosuppressed mice demonstrating disseminated infection with multi-organ involvement including orchitis effectively treated by recombinant type I interferons. EBioMedicine.

[30] Lazear HM, Govero J, Smith AM (2016). A mouse model of Zika virus pathogenesis. Cell Host Microbe.

[31] Rossi SL, Tesh RB, Azar SR (2016). Characterization of a novel murine model to study Zika virus. Am J Trop Med Hyg.

[32] Dudley DM, Newman CM, Lalli J (2017). Infection via mosquito bite alters Zika virus tissue tropism and replication kinetics in rhesus macaques. Nat Commun.

[33] Hirsch AJ, Smith JL, Haese NN, Broeckel RM, Parkins CJ, Kreklywich C, DeFilippis VR, Denton M, Smith PP, Messer WB, Colgin LMA, Ducore RM, Grigsby PL, Hennebold JD, Swanson T, Legasse AW, Axthelm MK, MacAllister R, Wiley CA, Nelson JA, Streblow DN (2017). Zika virus infection of rhesus macaques leads to viral persistence in multiple tissues. PLoS Pathog.

[34] Newman C, Friedrich TC, O'Connor DH (2017). Macaque monkeys in Zika virus research: 1947-present. Curr Opin Virol.

[35] Kublin Jessica L., Whitney James B. (2018). Zika virus research models. Virus Research.

[36] Osuna CE, Whitney JB (2017). Nonhuman primate models of Zika virus infection, immunity, and therapeutic development. J Infect Dis.

[37] Chan JF, Yip CC, Tsang JO (2016). Differential cell line susceptibility to the emerging Zika virus: implications for disease pathogenesis, non-vector-borne human transmission and animal reservoirs. Emerg Microbes Infect.

[38] Nagata M (2016). Podocyte injury and its consequences. Kidney Int.

[39] Pavenstädt H, Kriz W, Kretzler M (2003). Cell biology of the glomerular podocyte. Physiol Rev.

[40] Barisoni L, Mundel P (2003). Podocyte biology and the emerging understanding of podocyte diseases. Am J Nephrol.

[41] Peralta-Aros C, García-Nieto V (2017). Does Zika virus infection induce prolonged remissions in children with idiopathic nephrotic syndrome?. Pediatr Nephrol.

[42] Chimelli L, Melo ASO, Avvad-Portari E (2017). The spectrum of neuropathological changes associated with congenital Zika virus infection. Acta Neuropathol.

[43] Costa Monteiro LM, Cruz GNO, Fontes JM (2018). Neurogenic bladder findings in patients with congenital Zika syndrome: a novel condition. PLoS One.

[44] Nogueira ML, Estofolete CF, Terzian AC (2016). Zika virus infection and solid organ transplantation: a new challenge. Am J Transplant.

[45] Terzian ACB, Estofolete CF, Alves da Silva R (2016). Long-term viruria in Zika virus-infected pregnant women, Brazil, 2016. Emerg Infect Dis.

[46] Jia H, Zhang M, Chen M (2016). Zika virus infection in travelers returning from countries with local transmission, Guangdong, China, 2016. Travel Med Infect Dis.

[47] Tappe D, Pérez-Girón JV, Zammarchi L, Rissland J (2015). Cytokine kinetics of Zika virus-infected patients from acute to reconvalescent phase. Med Microbiol Immunol.

[48] Nazerai L, Schøller AS, Rasmussen POS, Buus S, Stryhn A, Christensen JP, Thomsen AR (2018). A new in vivo model to study protective immunity to Zika virus infection in mice with intact type I interferon signaling. Front Immunol.

[49] Andrade DV, Harris E (2017). Recent advances in understanding the adaptive immune response to Zika virus and the effect of previous flavivirus exposure. Virus Res.

